# Increased *Bacillus thuringiensis* δ-endotoxin Cry3Aa toxicity against longhorned beetle by fusing to peptide specifically binding to beetle Cx-cellulase

**DOI:** 10.1186/1753-6561-5-S7-P89

**Published:** 2011-09-13

**Authors:** Shu-Tang Zhao, Chang-Hua Guo, Jian-Jun Hu, Xiao-Jiao Han, Jun Chen, Meng-Zhu Lu

**Affiliations:** 1 State Key Laboratory of Forest Genetics and Tree Breeding, Research Institute of Forestry, Chinese Academy of Forestry, Beijing 100091, PR China; 2 International Centre for Bamboo and Rattan, Beijing 100102, PR China

## Background

*Bacillus thuringiensis* (Bt) Cry toxins have specific toxicity to susceptible insects. They are being used in transgenic plants or spray to control insect pests in agriculture [1, 2, 3]. Cry3A toxins are used extensively for biological control of coleopteran larvae [4, 5]. A Bt886-*Cry3Aa* gene that exhibited a high activity against Coleoptera insects isolated Our laboratory. Insect bioassay performed on *Anoplophora glabripennis* Motsch and *Apriona germari* Hope showed that the mortality of larvae fed with the product of this gene was over 60% [6]. However, both transgenic poplar with native *Cry3Aa* and withmodified-*Cry3Aa* by using poplar-prefered codons did little effects on longhorned beetles probably due to its low expression level in poplar.A peptide (LPPNPTK) named PCx that specifically bind to cellulase from midgut of longhorned beetle larvae was screened out from a phage display library previously in our laboratory[7].

## Materials and methods

Fused *Cry3Aa* genes with PCx coding sequence at 5’ or 3’-end were amplified using pET-30a(+)-*Cry3Aa* [6] as a template and designed primers, and. used to construct three recombinant plasmids (pET-30(+)-PCx*-Cry3Aa*, pET-30(+)*-Cry3Aa*-PCx and pET-30(+)*-Cry3Aa*). Target proteins were characterized by Western Blot, ELISA and LC-MS/MS methods. To analyze the activity of PCx-Cry3Aa, Cry3Aa-PCx and Cry3Aa proteins against longhorned beetle larvae, bioassays were performed on *A. germari*Hope larvae by artifical feed with toxins. The retaining time of target proteins in midgut and the cellulase activity of longhorned beetle larvae were measured in order to elucidate the potential mechanism of the fused toxins of PCx and Cry3Aa against longhorned beetle larvae.

## Results

**Figure 1:**
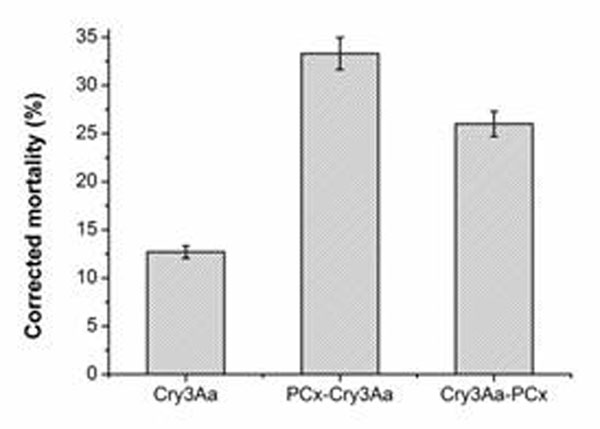
Toxicity of Cry3Aa and the Cry3Aa fusion proteins against *A. germari*Hope larvae. Corrected mortality rates are shown for larvae fed with Cry3Aa, PCx-Cry3Aa, or Cry3Aa-PCx. Both Cry3Aa fusion proteins exhibited increased toxicity toward the larvae, compared with non-modified Cry3Aa.

**Figure 2:**
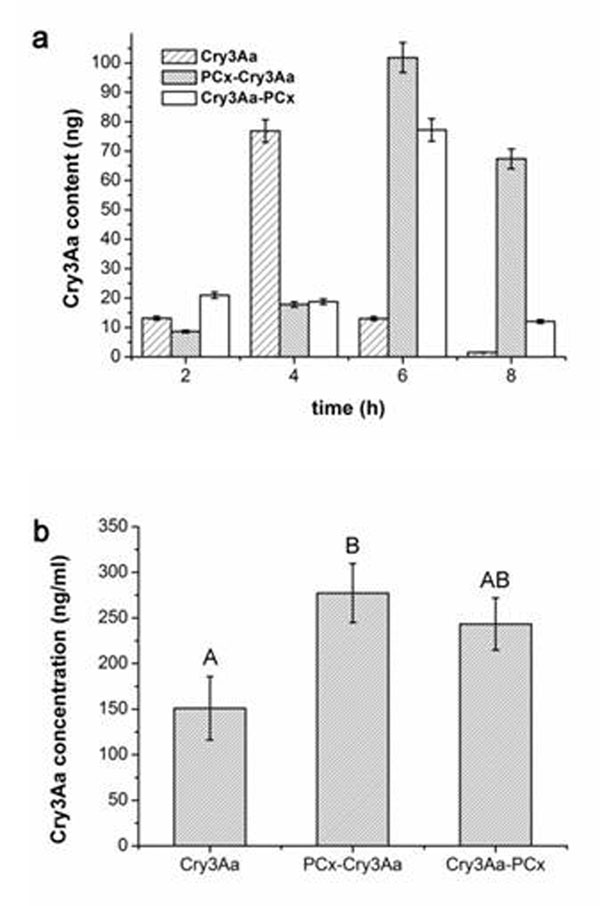
Concentration of Cry3Aa in *A. germari* Hope larvae after feeding with Cry3Aa and the Cry3Aa fusion proteins. a, Cry3Aa was measured in larval excretions collected at 2, 4, 6, and 8 h after feeding. The Cry3Aa content peaked at 4 h after feeding with Cry3Aa, whereas the peak was observed at 6 h after feeding with the Cry3Aa fusion proteins. b, Cry3Aa was measured in larval midgut extractions at 4 h after feeding. The concentration of Cry3Aa in the midgut extracts was higher after feeding with the fusion proteins, compared with non-modified Cry3Aa.

Expression products contained target proteins were characterized by SDS-PAGE, Western-blot and ELISA after induced by IPTG .The target bands were digested and analyzed by LC-MS/MS to further confirm them as Cry3Aa proteins. The bioassay showed that the mortality of larvae fed with the two fused Cry3Aa proteins (PCx*-Cry3Aa* and *Cry3Aa*-PCx) was up to three times higher than that fed with Cry3Aa (Figure 1). Retaining time analysis was performed on excretas that collected at different times after feeding. The result showed that the fused Cry3Aa was concentrated in excreta collected at 6 h, whereas Cry3Aa at 4 h. Meanwhile, the Cry3Aa concentration in midgut juice after fed with fused Cry3Aa was higher than that with Cry3Aa alone. This indicated that the retaining time of fused PCx-Cry3Aa in midgut of larvae is longer compared to that of Cry3Aa alone (Figure 2). In addition, we also analyzed the cellulase activity when bond with fused Cry3Aa or Cry3Aa alone and showed that the fused protein did not affect the activity of cellulase. Therefore, the remainning time of fused Cry3Aa is prolonged after binding with cellulase, thus the enhanced toxicity of fused Cry3Aa is due to the prolonged retaining time.

## Conclusions

These data demonstrate that the cellulase-binding peptide could enhance the toxicity of *Bacillus thuringiensis* Cry3Aa against the longhorned beetle.We also confirmed that the increased lethality in larvae fed with PCx-Cry3Aa or Cry3Aa-PCx was attributable to the ability of the toxin to bind Cx-cellulase, thereby increasing toxin retention in the midgut. These uniquely modified Cry3Aa proteins have potential use for pest control. The significantly enhanced activity of Cry3Aa fused with the Cx-cellulase-binding peptide provides a new strategy for increasing δ-endotoxin efficacy against the longhorned beetle.

